# Supplementary material for: Mass Spectrometric Characterization of Epoxidized Vegetable Oils
Source: Polymers (Basel). 2019 Feb 28;11(3):394. doi: 10.3390/polym11030394 (PMC6473735; doi:10.3390/polym11030394)
Supplement: Supplementary file 1 [file polymers-11-00394-s001.pdf]

Supplementary Materials

Mass Spectrometric Characterization of Epoxidized Vegetable Oils

Ákos Kuki, Tibor Nagy, Mahir Hashimov, Stella File, Miklós Nagy, Miklós Zsuga and Sándor Kéki*

Table of contents

FIG. S1 2
FIG. S2 2
FIG. S3 3
FIG. S4 4
FIG. S5 5
FIG. S6 6

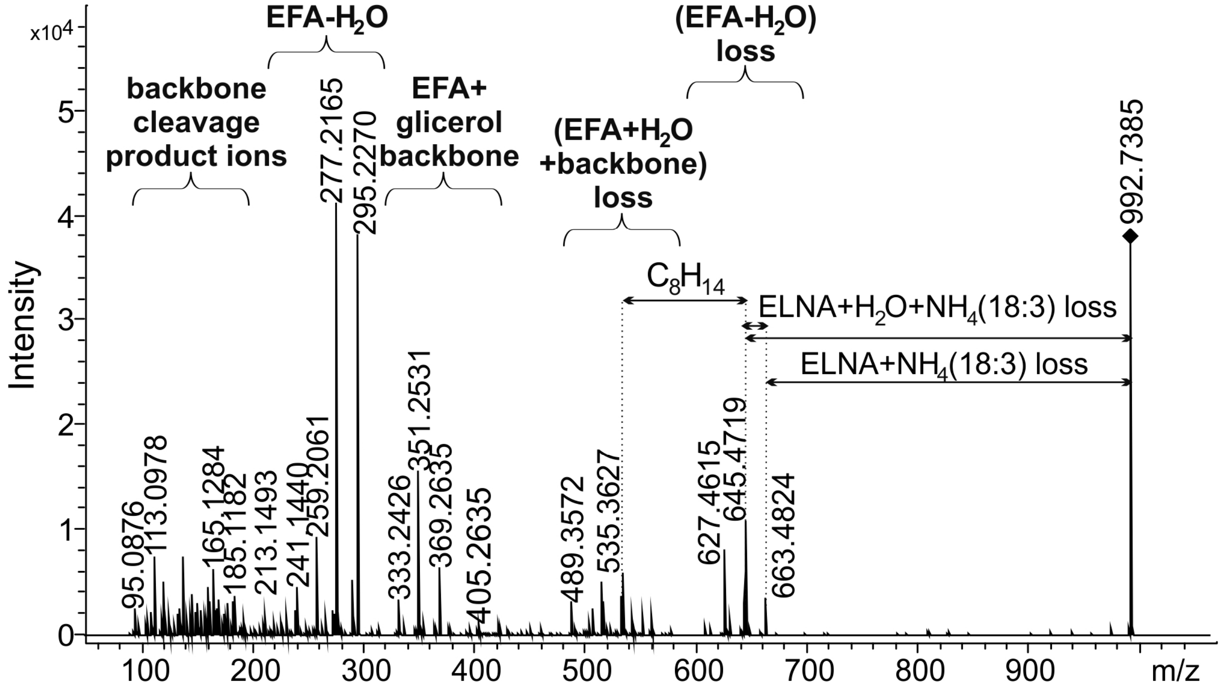

Figure S1.

ESI-QTOF MS/MS spectrum of the ammoniated (57:6) ETG adduct at *m/z* 992 of the soybean oil recorded at a laboratory frame collision energy of 36 eV. Abbreviations: EFA, epoxidized fatty acid; ELNA, epoxidized linolenic acid (18:3).

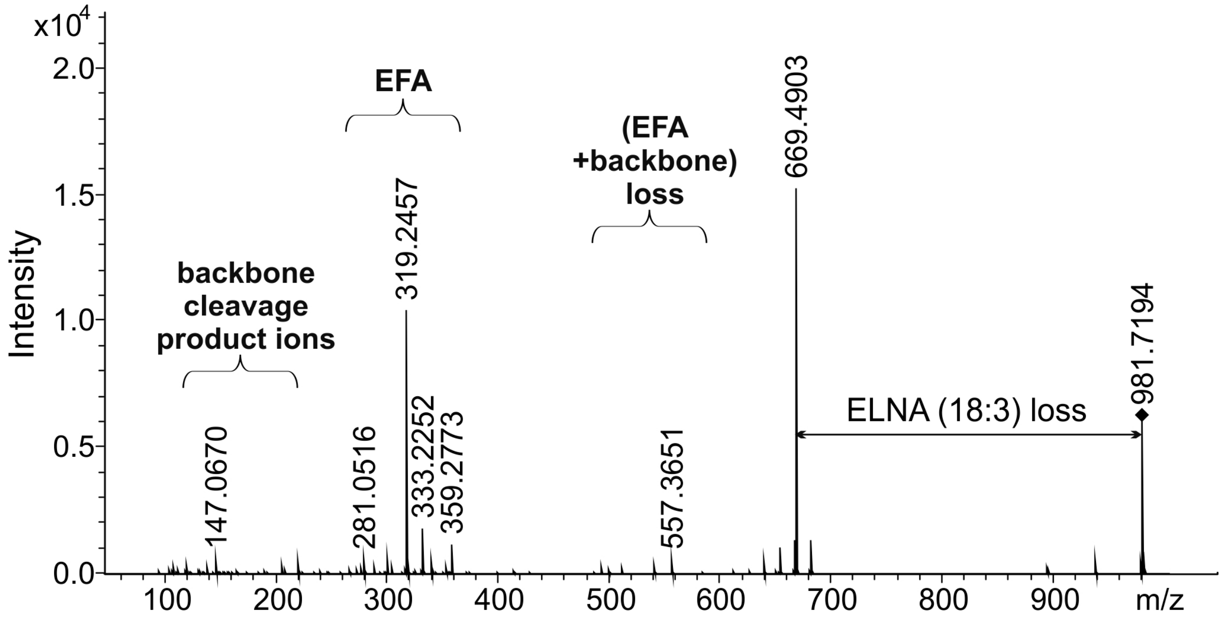

Figure S2

ESI-QTOF MS/MS spectrum of the lithiated (57:6) ETG adduct at *m/z* 981 of the soybean oil recorded at a laboratory frame collision energy of 65 eV. Abbreviations: EFA, epoxidized fatty acid; ELNA, epoxidized linolenic acid (18:3).

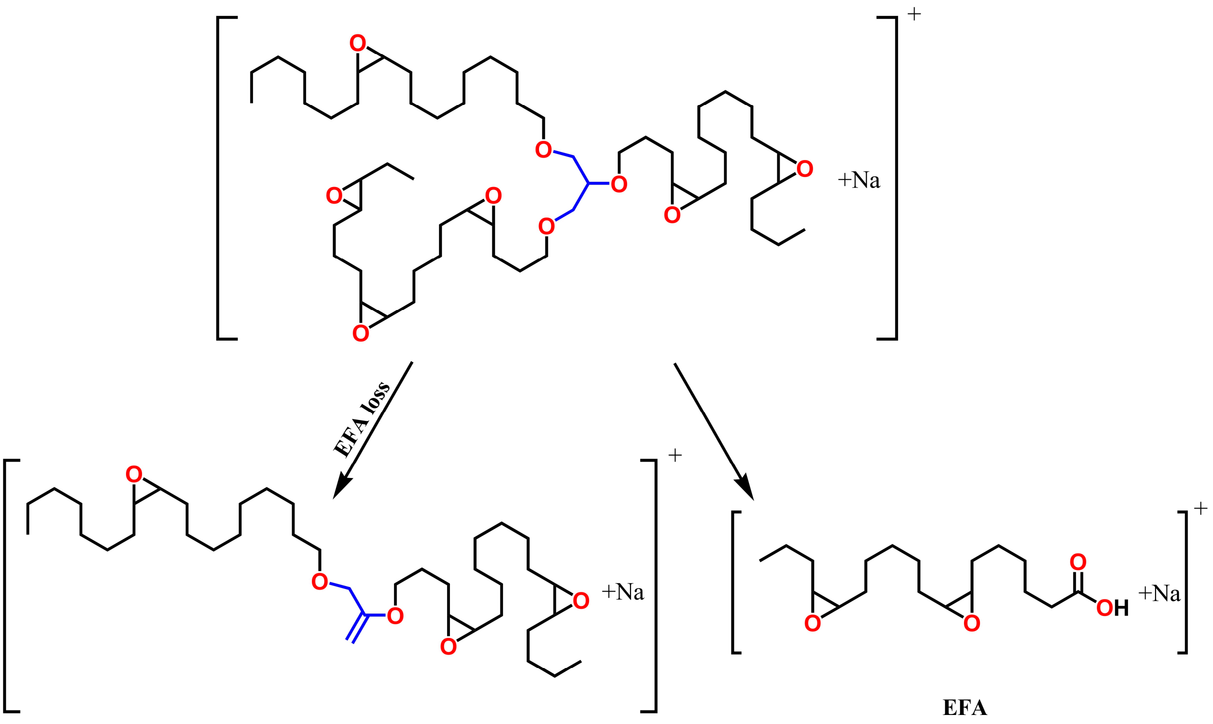

Figure S3

Proposed fragmentation pathways of the sodiated epoxidized triglyceride adduct. EFA stands for epoxidized fatty acid.

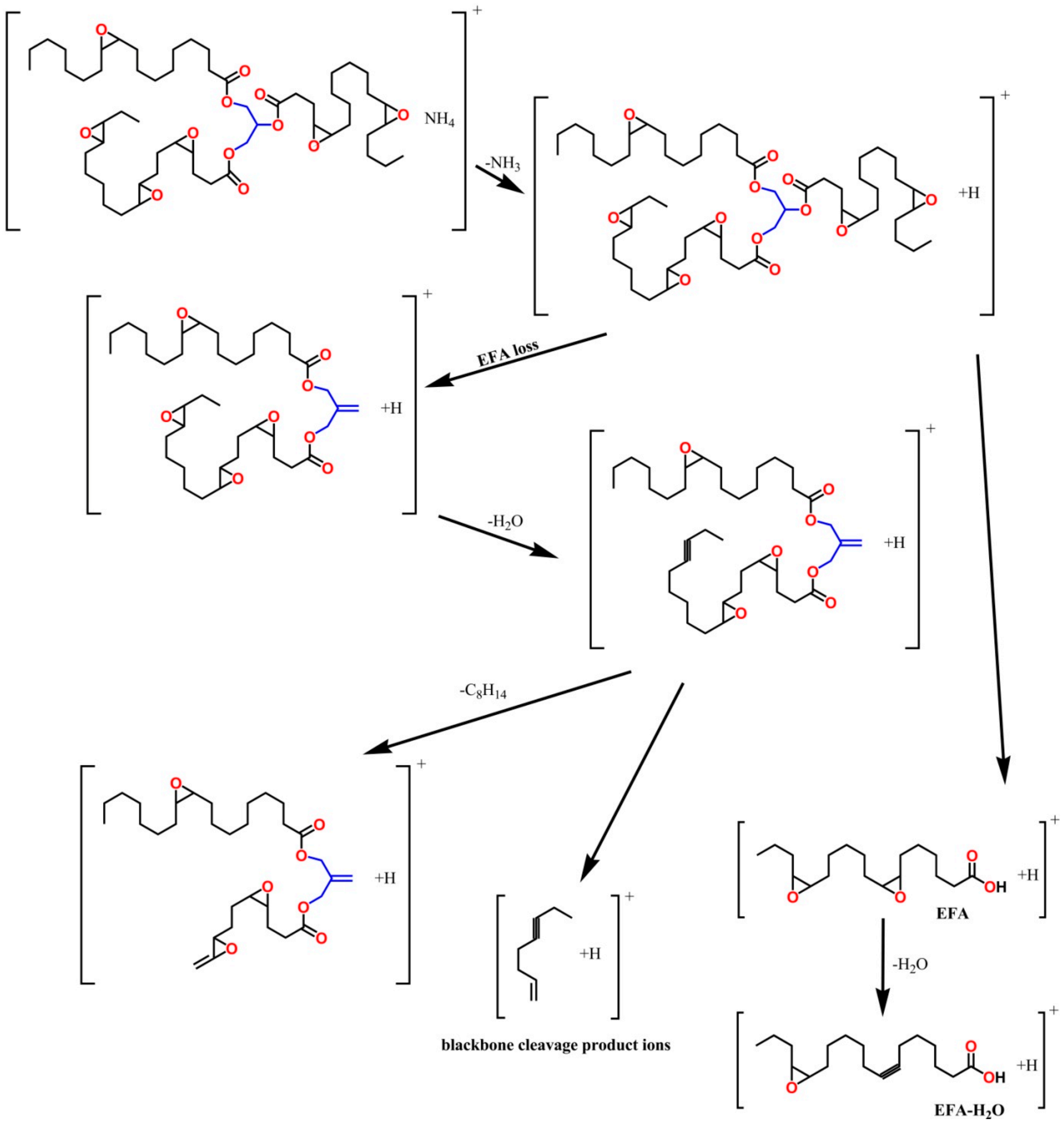

Figure S4

Proposed fragmentation pathways of the ammoniated epoxidized triglyceride adduct. EFA stands for epoxidized fatty acid.

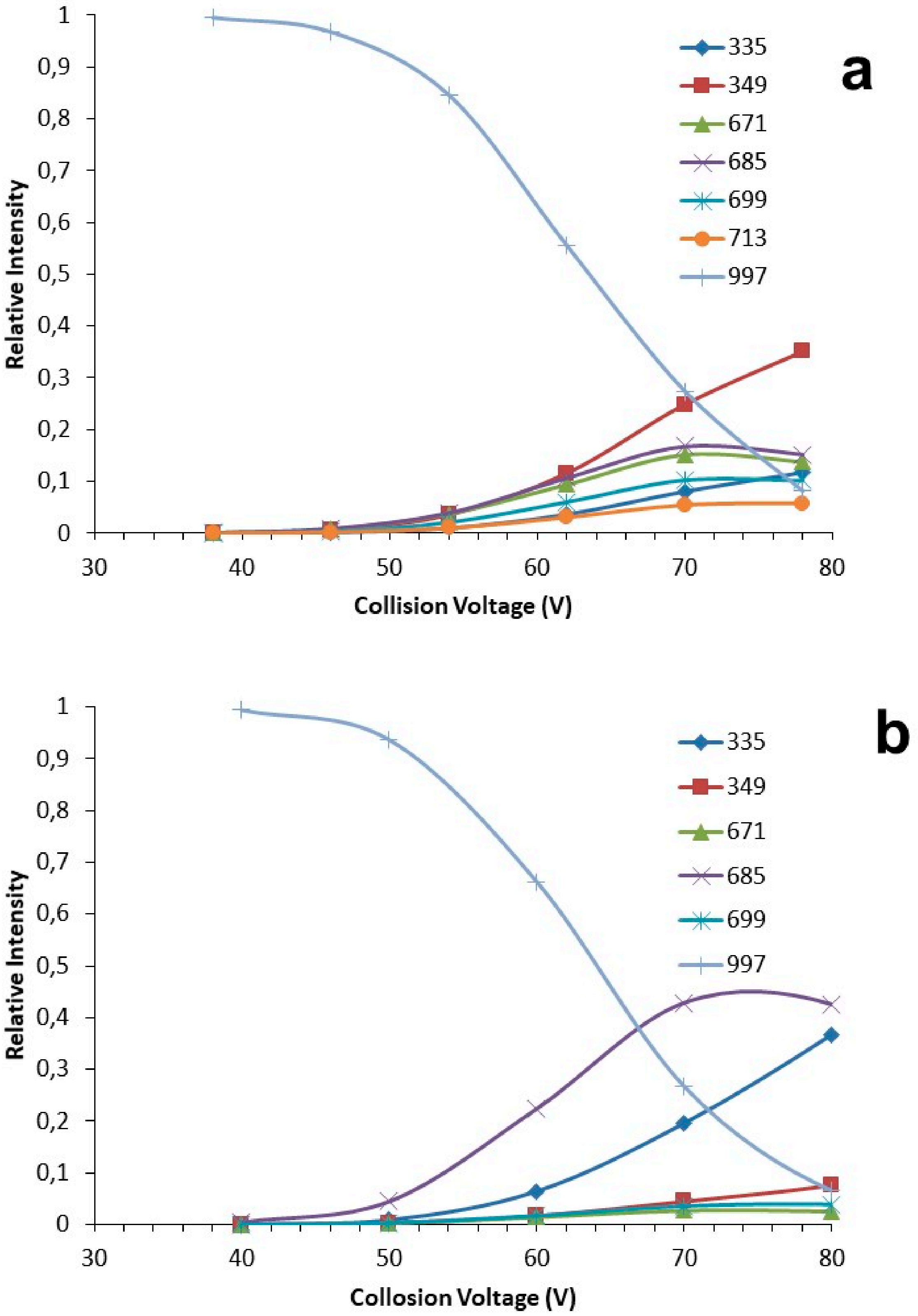

Figure S5

Breakdown diagrams for the fragment ions *m/z* 335, *m/z* 349, *m/z* 671, *m/z* 685, *m/z* 399, *m/z* 713, and the precursor ion *m/z* 997 of the sodiated epoxidized triglyceride (57:6) of the (**a**) linseed oil and (**b**) soybean oil. Relative intensity was calculated as the ratio of the intensity of a given product ion to that of all the product plus the precursor ions.

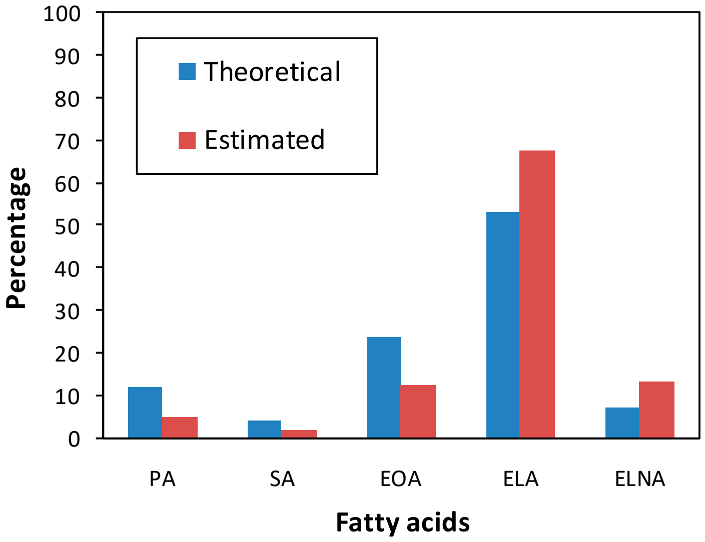

Figure S6

Epoxidized fatty acid composition of the epoxidized soybean oil calculated by our approach compared to the theoretical values (see in Ref. [1]). Abbreviations: PA, palmitic acid, (16:0); SA, stearic acid (18:0); EOA, epoxidized oleic acid, (18:1); ELA, epoxidized linoleic acid (18:2); ELNA, epoxidized linolenic acid (18:3).

Reference

1. Güner, F.S.; Yağcı, Y.; Tuncer Erciyes, A. Polymers from triglyceride oils. *Prog. Polym. Sci.* **2006**, *31*, 633–670.
